# Supplementary material for: Curation, inference, and assessment of a globally reconstructed gene regulatory network for Streptomyces coelicolor
Source: Sci Rep. 2022 Feb 18;12:2840. doi: 10.1038/s41598-022-06658-x (PMC8857197; doi:10.1038/s41598-022-06658-x)
Supplement: Supplementary file 4 — Supplementary Legends. [file 41598_2022_6658_MOESM4_ESM.docx]

Supplementary Information

• Supplementary File 1.docx: Word file with the Supplementary figures 1 – 14. NDA and structural analysis of the Curated_FL-DBSCR-RTB network. Supplementary methods and references.

• Supplementary File 2.xlsx: Excel file with Supplementary tables 1 – 10.

• Supplementary File 3.xlsx: Compressed folder with all the flat Files of the inferred networks.
